# Lung Cancer Treatment: From Tradition to Innovation

**DOI:** 10.3389/fonc.2022.858242

**Published:** 2022-05-27

**Authors:** Giuseppe Mangiameli, Ugo Cioffi, Alberto Testori

**Affiliations:** ^1^ Division of Thoracic Surgery, IRCCS Humanitas Research Hospital, Milan, Italy; ^2^ Department of Biomedical Sciences, Humanitas University, Milan, Italy; ^3^ Department of Surgery, University of Milan, Milan, Italy

**Keywords:** lung cancer, open lobectomy, VATS lobectomy, RATS lobectomy, thoracic surgery

## Abstract

Lung cancer (LC) is the second most commonly diagnosed cancer and the primary cause of cancer death worldwide in 2020. LC treatment is associated with huge costs for patients and society; consequently, there is an increasing interest in the prevention, early detection with screening, and development of new treatments. Its surgical management accounts for at least 90% of the activity of thoracic surgery departments. Surgery is the treatment of choice for early-stage non-small cell LC. In this article, we discuss the state of the art of thoracic surgery for surgical management of LC. We start by describing the milestones of LC treatment, which are lobectomy and an adequate lymphadenectomy, and then we focus on the traditional and innovative minimally invasive surgical approaches available: video-assisted thoracoscopic surgery (VATS) and robotic-assisted thoracoscopic surgery (RATS). A brief overview of the innovation and future perspective in thoracic surgery will close this mini-review.

## Introduction

Lung cancer (LC) is the second most commonly diagnosed cancer and the primary cause of cancer death worldwide in 2020 (1). These epidemiological data explain the growing interest in the prevention, early detection by screening programs, and development of new treatments for LC (2–4).

Whereas small cell LC (SCLC) patients are rarely treated by surgery, early-stage patients affected by non-SCLC (NSCLC) typically undergo surgery for resection and cure (2).

In this article, we discuss the state of the art of thoracic surgery in the surgical management of early NSCLC. We start by describing the principles of surgical therapy that should remain the milestones of NSCLC treatment: lobectomy and adequate lymphadenectomy (5, 6). Across time and under the impulse of technological innovation, different approaches have been developed from classical thoracotomy to minimally invasive surgery. Actually, the most common minimally invasive approaches are video-assisted thoracoscopic surgery (VATS) and robotic-assisted thoracoscopic surgery (RATS). These approaches have been developed in order to improve short-term outcomes, minimize pain, and maintain the same oncological results. In this mini-review, we describe several aspects of both open and minimally invasive surgery approaches, and we conclude with a brief overview of the future perspectives in thoracic surgery.

## Principles of Resection Surgery

One of the most important principles in oncological lung thoracic surgery is the multidisciplinary discussion of surgical cases. Several studies have shown that the best oncological outcomes are directly associated with multidisciplinary approaches (7). As a consequence, LC should be exclusively treated in centers with a multidisciplinary team composed of healthcare professionals dedicated to lung disease. Surgical volume and hospital volume are other principles that should be respected in the oncological and surgical management of LC. Several studies have shown that patients undergoing NSCLC resection in hospitals that perform a large number of such procedures can survive longer than patients who undergo such surgery in hospitals with a low volume of lung resection procedures. Likewise, the number of procedures performed by the surgeon is an important factor capable of predicting a better surgical outcome (8). Thus, only thoracic surgeons who perform LC surgery in highly specialized centers should be concerned about this surgery.

Surgery is the preferred local treatment modality that should be proposed for patients affected by early-stage NSCLC (9). LC surgery should be performed according to rigorous principles:

1. In all patients affected by NSCLC scheduled for surgery, an anatomic pulmonary resection should be preferred in the majority of cases. Lobectomy is the gold standard anatomic resection for NSCLC in patients who can tolerate the size of lung resection.
2. Sub-lobar resections (SLRs) such as segmentectomy or wedge resection should be performed in selected patients having a poor pulmonary reserve or other major comorbidities that contraindicate lobectomy or in peripheral nodules ≤2 cm presenting specific characteristics (pure histology, ≥50% ground-glass appearance on CT, or a long doubling time (≥400 days) confirmed by radiologic surveillance). However, in these cases, segmentectomy should be preferred to wedge resection.
3. Adequate lymphadenectomy is a fundamental part of NSCLC surgery. Hilar (N1) and mediastinal (N2) node resection and mapping should be routine components of LC resections. A minimum of three N2 stations should be sampled, or a complete mediastinal lymph node dissection (MLND) should be performed (6).

Interestingly, the scientific community continued to explore the role of SLR such as wedge resection or segmentectomy in treating LC. Indeed several retrospective studies demonstrated that SLR compared to lobectomy has no difference in overall survival or disease-free survival when performed in sub-centimeter tumors even in patients with good pulmonary function (9, 10, 11).

However, it should be clarified that in the literature there is evidence showing that, in treating small-sized NSCLCs, segmentectomy has been considered superior to wedge resections in the prognosis of early-stage NSCLC (12–14). Furthermore, some authors reported that overall and LC-specific survivals were significantly better among patients who underwent segmentectomy than with wedge resection in many stages of IA NSCLC (15). Nevertheless, two prospective randomized trials are currently ongoing (16) in order to provide evidence for the role of SLR in comparison to lobectomy. In the same manner, even if in current surgical guidelines the role and the extension of the lymphadenectomy are currently available, an accurate lymphadenectomy is not always performed. Several studies showed that patients randomized to complete MLND have less additional postoperative morbidity as compared with those undergoing random LN sampling (LNS), and generally, MLND does not increase the length of stay (17–21). Furthermore, about 15% of pN+ patients had mediastinal LN metastasis that did not follow a lobe-specific lymphatic diffusion, justifying a radical dissection of mediastinal nodes to avoid misdiagnosis of metastatic nodes, not lobe-specific lymphatic stations (22).

In conclusion, definitive management of LC for a cure necessitates anatomical resection of the entire involved lobe with hilar and MLND in patients who can tolerate the resection. Thus, performing a complete MLND is relatively harmless and low risk and remains the best “sampling” even in clinical stage I NSCLC to correctly stage patients and to offer them an evidence-based adjuvant therapy and, furthermore, an R0 resection.

## Open Surgery

Lobectomy, defined as the surgical removal of the entire lobe of the lung, has traditionally been performed through a thoracotomy approach. The outcome of the procedure is largely dependent on patient selection. Patients with forced expiratory volume in 1 s (FEV1) of less than 800 cm^3^ or diffusion capacity of carbon monoxide (DLCO) of less than 40% are considered high-risk patients. In these patients, sub-lobar resection or non-operative therapy should be proposed (9). Rib-spreading thoracotomy has been the standard procedure since the first endeavors in thoracic surgery. So for conventional lobectomy procedures, a rib retractor is the mainstay surgical tool (23). Thoracotomy provides excellent exposure to the pulmonary hilum and allows direct two-handed surgical techniques for exposure, retraction, and sharp dissection. Different incisions to access the pleural cavity have been standardized through time by different surgical groups. **Table 1** shows all the most common thoracotomies performed in lung surgery and their relative advantages and disadvantages.

**Table 1:** All most common thoracotomies performed in lung surgery and relative advantages and disadvantages.

	Site	Patient’s position	Advantage	Disadvantages
**Postero-lateral**	From a point located at 3 inches from the mid-spinal line to the anterior axillary line, passing below the tip of the scapula	Lateral decubitus	- Excellent exposure - Muscle-sparing variations are possible	Transection of large muscles: - increased potential for blood loss - prolonged ipsilateral shoulder and arm dysfunctions - compromised pulmonary function - chronic post-thoracotomy pain
**Lateral**	Horizontal line passing below the tip of the scapula to the sub-mammary fold	Lateral decubitus	- Excellent exposure - Latissimus dorsi muscle integrity is retained	- An extension to the upper ribs cannot be safely performed
**Anterior**	Lateral edge of the sternum and curves along the sub-mammary crease to the anterior axillary line	Ipsilateral side elevated approximately 30° to 45°	- Excellent access to the upper lobe, the right middle lobe, or the anterior hila -Superior cosmetic results	- Limited exposure to the posterior pleural space

In performing any kind of thoracotomy, it is important to choose the most appropriate and least traumatic surgical incision, adhere to meticulous surgical techniques, and avoid intercostal nerve injury or rib fractures. Unfortunately, rib fracture is a common occurrence during thoracotomy; thus, the rib spreader should be progressively and slowly opened to minimize the risk of fracture. In order to prevent fracturing, the ribs may be intentionally divided or “shingled” posteriorly at the costovertebral angle or anteriorly at costo-chondral articulations according to the type of performed thoracotomy (24).

In conclusion, the first principle in making a thoracic incision is that adequate exposure must be achieved, especially during the most technically challenging part of the operation. The second principle is that chest-wall function and appearance should be preserved to the extent possible. The choice of incision is aided by a thorough understanding of the surface anatomy and a comprehensive review of the radiographic images that are obtained preoperatively. Finally, independent of the chosen approach, the oncological results after anatomical open lobectomy (OL) for the early-stage NSCLC are good. The completeness of resection, stage, and LN involvement are the primary predictors of survival after resection. The 5-year overall survival rate was reported to range between 73.8% and 78.9% (25). Furthermore, OL is usually associated with a significant risk of postoperative complications occurrence. Depending on the surgical series, up to 35% of patients may experience some form of postoperative complication after an OL. The most common of these are minor and include atrial arrhythmia and prolonged air leak, but more serious complications including respiratory failure can occur and increase in frequency with decreased baseline pulmonary function. The operative mortality following lobectomy is reported to be 1% to 3%, with pneumonia and respiratory failure as the overwhelming causative factors (26).

To date, stage I NSCLC is still treated by OL, but in the coming years, minimally invasive approaches will probably overcome this traditional approach.

## Video-Assisted Thoracoscopic Surgery

VATS has been the first non-rib-spreading thoracic procedure described. It differs from a mini-thoracotomy by the lack of rib spreading and complete thoracoscopic visualization as opposed to visualizing the procedure directly through the incisions (27). Through time, many studies have reported a higher incidence of morbidity and less favorable outcomes when thoracotomy is compared to non-rib-spreading procedures (28). The neuralgic pain caused by irritation of the intercostal nerves, which is naturally exacerbated by rib spreading, is the leading cause of postoperative morbidity after thoracotomy. It usually leads to poor respiratory effort and subsequent atelectasis or pneumonia. The advantages of the non-rib-spreading technique are immediately related to a reduction of acute postoperative pain as reported in several studies, as after no spreading procedure a lower amount of medication is required, and usually, a higher proportion of patients present a very low postoperative pain profile. Furthermore, several studies confirmed a reduction in the occurrence of chronic pain and a longer and better quality of life when thoracotomy was compared to the non-rib-spreading technique (29).

Initial thoracoscopic procedures were reported in the early 20th century (30), but the widespread use of the VATS technique did not occur until the 1980s with improvement in video technology, the introduction of double-lumen endotracheal tubes, and the mechanical surgical stapler allowing to securely divide pulmonary parenchyma, bronchi, and vessels through small incisions (31–33).

The first VATS lobectomy reports emerged in the 1990s, documenting the safety and outlining technical aspects of the approach (34). The VATS approach to lobectomy for NSCLC typically involves a varying number of small incisions (two- to four-port sites) and a 5- to 8-cm access incision (utility port). To date, various approaches to performing VATS lobectomy have been reported in the literature (see **Table 2**) (23, 35–39). Nevertheless, the posterior VATS approaches never really became widely performed, and most centers use a utility incision measuring about 3–5 cm generally positioned anteriorly with one or two adjunctive ports (40). Uni-portal VATS is also an adopted technique to perform lobectomy with a long incision ranging from 2.5 to 5 cm, which should be selected according to patients’ chest size, the lobe of the lung, the diameter of the tumor, and body mass index (41–43).

**Table 2:** Minimally invasive surgical techniques to perform lobectomy.

VATS	Characteristics
**Anterior approach**	(I) 4th intercostal space utility incision is directly over the hilum (to easily clamp the major vessels in case of the major bleeding) (II) The surgeon does not need to change his/her position or the site of incision if a conversion is required (III) Major vessels are the first structures to be transected (IV) Reproducibility
Copenhagen, three ports (34)	
D’Amico, two ports (35)	
Gonzales-Rivas, uni-port (36)	
**Posterior approach**	(I) The surgeons are placed posterior to the patient (II) Utility incision is made at the 6th or 7th intercostal space anterior to latissimus dorsi muscle (III) Camera port is made through the auscultatory triangle, instead of lower anterior incision (IV) Thoracoscopy is 0° rather than 30° (V) The order of dissection is from the posterior to anterior, by opening up the fissure first to identify and isolate pulmonary arterial branches
Edinburgh, three ports posterior approach (37)	
**Purely thoracoscopic lobectomies** (38)	(I) No utility incisions (II) Completely portal procedure
**RATS**	**Advantages compared to VATS**
**Utility thoracotomy without capnothorax**	(I) Binocular visualization: excellent high-definition three-dimensional view of the operating field (fine dissection with precision and accuracy) (II) Robotic instruments have a greater precision, a superior range of motion (degrees of freedom), and improved ergonomic characteristics (III) Motion scaling and zoom capabilities
Park (three arms) (39)	
Veronesi (fours arms) (40)	
**Completely portal with capnothorax**	(IV) Carbon dioxide insufflation favoring further collapse of the lung provides a larger working area.
Cerfolio (fours arms) (41)	
Cerfolio-modified techniques (42)	

VATS, video-assisted thoracoscopic surgery; RATS, robotic-assisted thoracoscopic surgery.

Independent of the chosen approach, VATS lobectomy has the same oncological operation as the open approach, with the removal of the pulmonary lobe containing the tumor with individual ligation of each of the bronchovascular structures and removal of hilar and mediastinal LNs. In the past decade, numerous large series have reported recurrence and survival data that are equivalent to OL. Furthermore, the largest series of lobectomy by VATS describe a similar pattern of perioperative complications as the open approach but at reduced rates (44). Other consistently demonstrated advantages of VATS lobectomy over OL are earlier recovery, better quality of life, increased delivery of adjuvant therapy, less impact on pulmonary function tests and the immune system, decreased pain, and reduced length of stay (45).

Thus, according to several guidelines, VATS or minimally invasive surgery (including RATS) should be strongly considered for patients with no anatomic or surgical contraindications, as long as there is no compromise of standard oncological and dissection principles of thoracic surgery (6, 46).

However, despite the undoubted advantages, VATS lobectomy spreading is not ubiquitous. It is currently estimated that the VATS lobectomy rate is 30%–40% in the United States, 30% in Europe, 50% in Italy, 65% in Denmark, and 29% in Great Britain and Ireland (47). Several factors can explain the slow transition from thoracotomy to video-assisted surgery, despite the obvious advantages. One of these is surely the demanding learning curve and skill acquisition to face unexpected intraoperative complications, such as bleeding. Another explication is that VATS has become the technique of choice in the early stages of LC, which actually account for about 25% of surgical NSCLC cases.

## Robotic-Assisted Thoracoscopic Surgery

The introduction of RATS is undoubtedly the most recent significant addition to the field of thoracic surgery that, as an “innovative technological bomb,” has changed the entire paradigm of the traditional approach to surgery. Early experience with da Vinci robots (Intuitive Surgical, Sunnyvale, CA, USA) showed that this minimally invasive approach is feasible and safe (48). The spreading of RATS results from having aspects comparable to but, at the same time, has several advantages as compared to VATS. Like VATS, RATS allows anatomical thoracic resections through smaller non-rib-spreading incisions resulting in less operative trauma for the patient. All advantages typical of RATS are reported in **Table 2**.

The current approach for a robotic lobectomy consists of similar lateral decubitus positioning as the open or VATS approach. The main difference compared to open or VATS lobectomy is that during a RATS procedure, the surgeon is not at the bedside and not even sterile. The surgeon controls a three-dimensional (3D) high-definition camera and instruments that can fit through 8-mm ports from a remote console (48).

A robotic system was used for the first time in performing a lobectomy for treating primary NSCLC in 2002 (49). From this first report, several surgical series have confirmed that this minimally invasive approach is safe. Different techniques of RATS lobectomy have been described and can be resumed in two groups: without insufflation with a utility thoracotomy or completely portal with carbon dioxide insufflation (capnothorax) (see **Table 2**) (50–53).

During RATS lobectomy, the hilar and fissural dissections are similar to those of VATS and open approaches. The bronchovascular structures are dissected and individually divided with staplers, as with other approaches. The stapler is usually introduced by utility incision or by assistant’s port. Finally, the specimen is generally removed from the chest through the utility incision or through a widened assistant’s port when a completely port-based robotic lobectomy is performed (53).

To date, no randomized controlled studies have compared the different surgical approaches of lung surgery: thoracotomy, VATS, and RATS. The initial series of patients undergoing robotic lobectomy for NSCLC demonstrate safety, feasibility, and similar morbidity and mortality rates compared with OL or VATS approaches (54). A recent prospective randomized control trial compared the perioperative outcome and surgical radicality of the robotic approach with those of traditional VATS in the treatment of early-stage NSCLC and confirmed that RATS was not superior to VATS considering the perioperative outcome for early-stage NSCLC, but the robotic approach allowed an improvement of LN dissection (55). Concerning the oncological benefits of robotic surgery, longer follow-up data should be awaited. At the same time, initial reports show comparable stage-specific survival rates between the VATS and robotic approaches (56). Probably the only limitation to a widespread diffusion of RATS is the high cost even if several studies have demonstrated that RATS appears to have an overall cost–benefit due to the significant decrease in length of hospital stay as compared to open surgery (48). It is estimated that in 2015 approximately 15% of the lobectomies were performed with a robotic system in the United States (57).

## Innovation and Future Perspective in Thoracic Surgery

Several innovations have been recently introduced and probably will support the spread of robotic surgery. They can be summed up in the following:

1. Introduction of robotic EndoWrist staplers. Stapler division of the hilar structures is considered one of the most important and potentially hazardous steps during a lobectomy. To date, with the Xi Da Vinci system, many surgeons needed to also use a 12-mm assistant port for stapling. For some surgeons, the delegation of this task to the assistant is considered a risk. With the introduction of the Xi Da Vinci System, a 12-mm port can be used for the introduction and firing of the robotic EndoWrist stapler. The introduction of the robotic stapler allows the surgeon to operate with absolute autonomy managing by themselves the vascular section safely and efficiently. The operating surgeon’s ability to control the stapler from the console represents a critical technical advancement, as it can allow a growing number of surgeons to explore RATS and perhaps to transition from open or video-assisted lobectomy to RATS in the near future (58).
2. Development of the “single-site” technology. It represents the first desirable goal for robotic thoracic surgery. A thoracic uni-portal dispositive is in development, and it is expected to be commercialized in a few years (59).
3. Diffusion of fluorescence-guided surgery. Fluorescence is a new technology that has evolved concurrently with robotics. Recently, a new optical system was created and incorporated into the da Vinci platform, and it can be utilized to perform fluorescence-guided surgery using intravenous administration of the indocyanine green, allowing identification of the intersegmental plane in anatomic lung segmentectomies (53).
4. Electromagnetic navigational bronchoscopy and real-time 3D imaging. Electromagnetic navigation bronchoscopy is a minimally invasive, image-guided approach to access peripheral lung lesions, allowing biopsy, staging, fiducial placement, and dye marking in a single procedure.
  In the near future, the use of intraoperative imaging and the development of real-time 3D imaging and real-time image-guided therapy combined with a navigation system could allow to minimize unnecessary resection of healthy lung tissue in frail patients. Several clinical applications of these innovative tools have already been described (60).
5. Commercialization of new robots. Some companies (Medtronic and Johnson & Johnson with Google) are about to commercialize new robots. Their placing on the market could reduce the costs related to this technology, produced until now by a single company, and improve its widespread use. In the same manner, the introduction of a technology capable of receiving tactile feedback could finally improve the adoption of RATS as a minimally invasive approach of choice considering that RATS allows for performing the same surgical maneuvers that a surgeon usually performs during open surgery (53).

## Conclusion

In conclusion, even today, lobectomy is the gold standard surgery that should be proposed for treating early-stage NSCLC in patients who can tolerate anatomical lung resection. An adequate lymphadenectomy is a fundamental part of NSCLC surgery; a complete MLND allows for correctly staged patients and offers them an evidence-based adjuvant therapy and an R0 resection. Open thoracotomy is actually the most common approach among surgical groups for treating early-stage NSCLC. At the same time, minimally invasive surgery is already being utilized and is increasingly adopted in surgical practice, allowing several advantages compared to OL (less pain and better quality of life) in maintaining the same rate of postoperative complications and of short- and long-term oncological results. Probably, RATS lobectomy, thanks to its technological widespread use and an expected cost reduction, will become a minimally invasive approach of choice in the near future.

## Author Contributions

All authors contributed equally to the manuscript and approved the final version.

## Conflict of Interest

The authors declare that the research was conducted in the absence of any commercial or financial relationships that could be construed as a potential conflict of interest.

## Publisher’s Note

All claims expressed in this article are solely those of the authors and do not necessarily represent those of their affiliated organizations, or those of the publisher, the editors and the reviewers. Any product that may be evaluated in this article, or claim that may be made by its manufacturer, is not guaranteed or endorsed by the publisher.

## References

[1] Sung H Ferlay J Siegel RL Laversanne M Soerjomataram I Jemal A. Global Cancer Statistics 2020: GLOBOCAN Estimates of Incidence and Mortality Worldwide for 36 Cancers in 185 Countries. CA Cancer J Clin (2021) 71(3):209–49. doi: 10.3322/caac.21660 33538338

[2] Crucitti P Gallo IF Santoro G Mangiameli G. Lung Cancer Screening With Low Dose CT: Experience at Campus Bio-Medico of Rome on 1500 Patients. Minerva Chir (2015) 70(6):393–9. 25700151

[3] Mangiameli G Longo F Grasso RF Iacopino A Rocco R Quintarelli F. Focus on Lung Cancer Screening Program at Campus Bio-Medico of Rome: Update on Over 3250 Patients. Minerva Chir (2017) 72(4):361–3. doi: 10.23736/S0026-4733.17.07336-9 28635245

[4] Infante M Chiesa G Solomon D Morenghi E Passera E Lutman FR. Surgical Procedures in the DANTE Trial, A Randomized Study of Lung Cancer Early Detection With Spiral Computed Tomography: Comparative Analysis in the Screening and Control Arm. J Thorac Oncol (2011) 6(2):327–35. doi: 10.1097/JTO.0b013e318200f523 21178639

[5] Alloisio M Infante M Cariboni U Testori A Soto Parra H Ravasi G. The Evolution of Surgery in non-Small Cell Lung Cancer. Tumori (2000) 86(5 Suppl 1):S42–6. doi: 10.1177/03008916000865s110 11195294

[6] National Comprehensive Cancer Network. NSCLC (Version 7.2021). Available at: https://www.nccn.org/professionals/physician_gls/pdf/nscl.pdf (Accessed December, 2021).

[7] Berghmans T Lievens Y Aapro M Baird AM Beishon M Calabrese F. European Cancer Organisation Essential Requirements for Quality Cancer Care (ERQCC): Lung Cancer. Lung Cancer (2020) 150:221–39. doi: 10.1016/j.lungcan.2020.08.017 33227525

[8] Bach PB Cramer LD Schrag D Downey RJ Gelfand SE Begg CB. The Influence of Hospital Volume on Survival After Resection for Lung Cancer. N Engl J Med (2001) 345(3):181–8. doi: 10.1056/NEJM200107193450306 11463014

[9] Hopstaken JS de Ruiter JC Damhuis RAM de Langen AJ van Diessen JNA Klomp HM. Stage I Non-Small Cell Lung Cancer: Treatment Modalities, Dutch Daily Practice and Future Perspectives. Cancer Treat Res Commun (2021) 28:100404. doi: 10.1016/j.ctarc.2021.100404 34058517

[10] Kodama K Doi O Higashiyama M Yokouchi H. Intentional Limited Resection for Selected Patients With T1 N0 M0 non-Small-Cell Lung Cancer: A Single-Institution Study. J Thorac Cardiovasc Surg (1997) 1143):347–53. doi: 10.1016/S0022-5223(97)70179-X 9305186

[11] Matsumura Y Yano M Yoshida J Koike T Kameyama K Shimamoto A. Early and Late Recurrence After Intentional Limited Resection for Ct1an0m0, non-Small Cell Lung Cancer: From a Multi-Institutional, Retrospective Analysis in Japan. Interact Cardiovasc Thorac Surg (2016) 23(3):444–9. doi: 10.1093/icvts/ivw125 27226401

[12] Okada M Koike T Higashiyama M Yamato Y Kodama K Tsubota N. Radical Sublobar Resection for Small-Sized Non-Small Cell Lung Cancer: A Multicenter Study. J Thorac Cardiovasc Surg (2006) 132(4):769–75. doi: 10.1016/j.jtcvs.2006.02.063 17000286

[13] Okada M Nishio W Sakamoto T Uchino K Yuki T Nakagawa A. Effect of Tumor Size on Prognosis in Patients With Non-Small Cell Lung Cancer: The Role of Segmentectomy as a Type of Lesser Resection. J Thorac Cardiovasc Surg (2005) 129(1):87–93. doi: 10.1016/j.jtcvs.2004.04.030 15632829

[14] Sienel W Dango S Kirschbaum A Cucuruz B Hörth W Stremmel C. Sublobar Resections in Stage IA Non-Small Cell Lung Cancer: Segmentectomies Result in Significantly Better Cancer-Related Survival Than Wedge Resections. Eur J Cardiothorac Surg (2008) 33(4):728–34. doi: 10.1016/j.ejcts.2007.12.048 18261918

[15] Smith CB Swanson SJ Mhango G Wisnivesky JP. Survival After Segmentectomy and Wedge Resection in Stage I non-Small-Cell Lung Cancer. J Thorac Oncol (2013) 8(1):73–8. doi: 10.1097/JTO.0b013e31827451c4 23164939

[16] Nakamura K Saji H Nakajima R Okada M Asamura H Shibata T. A Phase III Randomized Trial of Lobectomy Versus Limited Resection for Small-Sized Peripheral Non-Small Cell Lung Cancer (JCOG0802/WJOG4607L). Jpn J Clin Oncol (2010) 40(3):271–4. doi: 10.1093/jjco/hyp156 19933688

[17] Riquet M Pricopi C Mangiameli G Arame A Badia A Le Pimpec Barthes F. Adequacy of Intra-Operative Nodal Staging During Lung Cancer Surgery: A Poorly Achieved Minimum Objective. J Thorac Dis (2018) 10(3):1220–4. doi: 10.21037/jtd.2018.01.174 PMC5906224 29707270

[18] Riquet M Pricopi C Mangiameli G Arame A Badia A Le Pimpec Barthes F. Occult Pn2 Disease in Lung Cancer Patients: A Wide Range of Diseases Endangering the Long-Term Prognosis. J Thorac Dis (2017) 9(8):2271–5. doi: 10.21037/jtd.2017.07.23 PMC5594192 28932522

[19] Riquet M Pricopi C Mangiameli G Arame A Badia A Le Pimpec Barthes F. Pathologic N1 Disease in Lung Cancer: The Segmental and Subsegmental Lymph Nodes. J Thorac Dis (2017) 9(11):4286–90. doi: 10.21037/jtd.2017.10.119 PMC5721051 29268493

[20] Riquet M Arame A Pricopi C. Systematic Lymphadenectomy: 'Meta-Lysis' Does Not Need Meta-Analysis. Eur J Cardiothorac Surg (2017) 52(5):1011–2. doi: 10.1093/ejcts/ezx299 28950369

[21] Zhang J Mao T Gu Z Guo X Chen W Fang W. Comparison of Complete and Minimal Mediastinal Lymph Node Dissection for Non-Small Cell Lung Cancer: Results of a Prospective Randomized Trial. Thorac Cancer (2013) 4(4):416–21. doi: 10.1111/1759-7714.12040 28920232

[22] Riquet M Rivera C Pricopi C Arame A Mordant P Foucault C. Is the Lymphatic Drainage of Lung Cancer Lobe-Specific? A Surgical Appraisal. Eur J Cardiothorac Surg (2015) 47:543–9. doi: 10.1093/ejcts/ezu226 24875885

[23] Passera E Rocco G. From Full Thoracotomy to Uniportal Video-Assisted Thoracic Surgery: Lessons Learned. J Vis Surg (2017) 3:36. doi: 10.21037/jovs.2017.01.14 29078599 PMC5637876

[24] Iwasaki A Hamatake D Shirakusa T. Biosorbable Poly-L- Lactide Rib-Connecting Pins may Reduce Acute Pain After Thoracotomy. Thorac Cardiovasc Surg (2004) 52:49–53. doi: 10.1055/s-2004-815802 15002077

[25] Dziedzic DA Zbytniewski M Gryszko GM Cackowski MM Langfort R Orlowski TM. Video-Assisted Versus Open Thoracotomy Lobectomy: Comparison on Lymphadenectomy and Survival in Early Stage of Lung Cancer. J Thorac Dis (2021) 13(1):101–12. doi: 10.21037/jtd-20-2251 PMC7867811 33569190

[26] Ginsberg RJ Hill LD Eagan RT Thomas P Mountain CF Deslauriers J. Modern Thirty-Day Operative Mortality for Surgical Resections in Lung Cancer. J Thorac Cardiovasc Surg (1983) 86(5):654–8. doi: 10.1016/S0022-5223(19)39080-4 6632940

[27] Rocco G Internullo E Cassivi SD Van Raemdonck D Ferguson MK. The Variability of Practice in Minimally Invasive Thoracic Surgery for Pulmonary Resections. Thorac Surg Clin (2008) 18(3):235–47. doi: 10.1016/j.thorsurg.2008.06.002 18831498

[28] Lim E Batchelor T Shackcloth M Dunning J McGonigle N Brush T. VIOLET Trialists. Study Protocol for VIdeo Assisted Thoracoscopic Lobectomy Versus Conventional Open LobEcTomy for Lung Cancer, a UK Multicentre Randomised Controlled Trial With an Internal Pilot (the VIOLET Study). BMJ Open (2019) 9:e029507. doi: 10.1136/bmjopen-2019-029507 PMC6797374 31615795

[29] Bendixen M Jørgensen OD Kronborg C Andersen C Licht PB. Postoperative Pain and Quality of Life After Lobectomy *via* Video-Assisted Thoracoscopic Surgery or Anterolateral Thoracotomy for Early Stage Lung Cancer: A Randomised Controlled Trial. Lancet Oncol (2016) 17(6):836–44. doi: 10.1016/S1470-2045(16)00173-X 27160473

[30] Jacobaeus H. Ueber Die Zystotskopie Bei Untersuchung Seroser Hohlungen Anzuqwnden. Munch Med Wochenschr (1910) 57:2090–22092.

[31] Amosov NM Berezovsky KK. Pulmonary Resection With Mechanical Suture. J Thorac Cardiovasc Surg (1961) 41:325–35. doi: 10.1016/S0022-5223(20)31694-9 13683052

[32] Longo F Crucitti P Quintarelli F Rocco R Mangiameli G Rocco G. Bipolar Sealing Devices in Video-Assisted Thoracic Surgery. J Vis Surg (2017) 3:13. doi: 10.21037/jovs.2017.01.07 29078576 PMC5638387

[33] Miller DL Roy S Kassis ES Yadalam S Ramisetti S Johnston SS. Impact of Powered and Tissue-Specific Endoscopic Stapling Technology on Clinical and Economic Outcomes of Video-Assisted Thoracic Surgery Lobectomy Procedures: A Retrospective, Observational Study. Adv Ther (2018) 35(5):707–23. doi: 10.1007/s12325-018-0679-z PMC5960486 29663180

[34] Kirby TJ Mack MJ Landreneau RJ Rice TW. Initial Experience With Video-Assisted Thoracoscopic Lobectomy. Ann Thorac Surg (1993) 56(6):1248–52. doi: 10.1016/0003-4975(93)90661-z 8267420

[35] Hansen HJ Petersen RH. Video- Assisted Thoracoscopic Lobectomy Using a Standardized Three- Port Anterior Approach - The Copenhagen Experience. Ann Cardiothorac Surg (2012) 1(1):70–6. doi: 10.3978/j.issn.2225-319X.2012.04.15 PMC3741716 23977470

[36] Burfeind WR D’Amico TA. “Thoracoscopic Lobectomy.” In: Operative Techniques in Thoracic and Cardiovascular Surgery, vol. 9.2. p. 98–114.

[37] Gonzalez-Rivas D. VATS Lobectomy: Surgical Evolution from Conventional VATS to Uniportal Approach. Scientific World Journal. (2012) 2012:780842. doi: 10.1100/2012/780842 23346022 PMC3544256

[38] Richards JMJ Dunning J Oparka J Carnochan FM Walker WS. Video-Assisted Thoracoscopic Lobectomy: The Edinburgh Posterior Approach. Ann Cardiothorac Surg (2012) 1(1):61–9. doi: 10.3978/j.issn.2225-319X.2012.04.17 PMC3741709 23977469

[39] Gossot D Girard P Raynaud C Stern JB Caliandro R Validire P. Totally Endoscopic Major Pulmonary Resection for Stage I Bronchial Carcinoma: Initial Results. Rev Mal Respir (2009) 26(9):961–70. doi: 10.1016/s0761-8425(09)73331-5 19953042

[40] Testori A Perroni G Voulaz E Crepaldi A Alloisio M. Pulmonary Lobectomy After COVID-19. Ann Thorac Surg (2021) 111(3):e181-e182. doi: 10.1016/j.athoracsur.2020.08.004 32987024 PMC7518286

[41] Migliore M. Uniportal Video-Assisted Thoracic Surgery: Twentieth Anniversary. J Thorac Dis (2018) 10(12):6442–5. doi: 10.21037/jtd.2018.12.49 PMC6344731 30746185

[42] Migliore M Hirai K. Uniportal VATS: Comment on the Consensus Report From the Uniportal VATS Interest Group (UVIG. Of the European Society of Thoracic Surgeons. Eur J Cardiothorac Surg (2020) 57(3):612. 10.1093/ejcts/ezz221 31397477

[43] Migliore M Halezeroglu S Molins L Van Raemdonck D Mueller MR Rea F. Uniportal Video-Assisted Thoracic Surgery or Single-Incision Video-Assisted Thoracic Surgery for Lung Resection: Clarifying Definitions. Future Oncol (2016) 12(23s):5–7. doi: 10.2217/fon-2016-0370 27712092

[44] McKenna RJ Jr Houck W Fuller CB. Video-Assisted Thoracic Surgery Lobectomy: Experience With 1,100 Cases. Ann Thorac Surg, 2006812421–425. doi: 10.1016/j.athoracsur.2005.07.078 16427825

[45] Nwogu CE D'Cunha J Pang H Gu L Wang X Richards WG. VATS Lobectomy has Better Perioperative Outcomes Than Open Lobectomy: CALGB 31001, an Ancillary Analysis of CALGB 140202 (Alliance). Ann Thorac Surg (2015) 99(2):399–405. doi: 10.1016/j.athoracsur.2014.09.018 25499481 PMC5616174

[46] Howington JA Blum MG Chang AC Balekian AA Murthy SC. Treatment of Stage I and II non-Small Cell Lung Cancer: Diagnosis and Management of Lung Cancer, 3rd Ed: American College of Chest Physicians Evidence-Based Clinical Practice Guidelines. Chest (2013) 143(5 Suppl):e278S–313S. doi: 10.1378/chest.12-2359 23649443

[47] Guerrera F Olland A Ruffini E Falcoz PE. VATS Lobectomy vs. Open Lobectomy for Early-Stage Lung Cancer: An Endless Question—Are We Close to a Definite Answer? J Thorac Dis (2019) 11(12):5616–8. doi: 10.21037/jtd.2019.12.19 PMC6988071 32030283

[48] Novellis P Bottoni E Voulaz E Cariboni U Testori A Bertolaccini L. Robotic Surgery, Video-Assisted Thoracic Surgery, and Open Surgery for Early Stage Lung Cancer: Comparison of Costs and Outcomes at a Single Institute. J Thorac Dis (2018) 10(2):790–8. doi: 10.21037/jtd.2018.01.123 PMC5864633 29607150

[49] Melfi FM Menconi GF Mariani AM Angeletti CA. Early Experience With Robotic Technology for Thoracoscopic Surgery. Eur J Cardiothoracic Surg (2002) 21:864–8. doi: 10.1016/s1010-7940(02)00102-1 12062276

[50] Park BJ Flores RM Rusch VW. Robotic Assistance for Video-Assisted Thoracic Surgical Lobectomy: Technique and Initial Results. J Thorac Cardiovasc Surg (2006) 131:54–9. doi: 10.1016/j.jtcvs.2005.07.031 16399294

[51] Veronesi G Agoglia BG Melfi F Maisonneuve P Bertolotti R Bianchi PP. Experience With Robotic Lobectomy for Lung Cancer. Innovations (Phila. (2011) 6:355–60. doi: 10.1097/IMI.0b013e3182490093 22436769

[52] Cerfolio RJ. Total Port Approach for Robotic Lobectomy. Thorac Surg Clin (2014) 24:151–6. doi: 10.1016/j.thorsurg.2014.02.006 24780418

[53] Mangiameli G Durand M. Robotic Left Ventral Segmentectomy. JTCVS Tech. (2021) 8:205–7. doi: 10.1016/j.xjtc.2021.04.035 PMC8350876 34401855

[54] Novellis P Maisonneuve P Dieci E Voulaz E Bottoni E Di Stefano S. Quality of Life, Postoperative Pain, and Lymph Node Dissection in a Robotic Approach Compared to VATS and OPEN for Early Stage Lung Cancer. J Clin Med (2021) 10(8):1687. doi: 10.3390/jcm10081687 33920023 PMC8071041

[55] Veronesi G Abbas AE Muriana P Lembo R Bottoni E Perroni G. Perioperative Outcome of Robotic Approach Versus Manual Videothoracoscopic Major Resection in Patients Affected by Early Lung Cancer: Results of a Randomized Multicentric Study (ROMAN Study). Front Oncol (2021) 11:726408. doi: 10.3389/fonc.2021.726408 34568057 PMC8458770

[56] Park BJ Melfi F Mussi A Maisonneuve P Spaggiari L Da Silva RK. Robotic Lobectomy for Non-Small Cell Lung Cancer (NSCLC): Long-Term Oncologic Results. J Thorac Cardiovasc Surg (2012) 143(2):383–9. doi: 10.1016/j.jtcvs.2011.10.055 22104677

[57] Terra RM Leite PHC Dela Vega AJM. Global Status of the Robotic Thoracic Surgery. J Thorac Dis (2021) 13(10):6123–8. doi: 10.21037/jtd-19-3271 PMC8575837 34795963

[58] Daryl PP. Robotic Lobectomy Utilizing the Robotic Stapler. Ann Thorac Surg (2016) 102:e591-3. doi: 10.1016/j.athoracsur.2016.05.105 27847093

[59] Konstantinidis KM Hirides P Hirides S Chrysocheris P Georgiou M. Cholecystectomy Using a Novel Single-Site(®) Robotic Platform: Early Experience From 45 Consecutive Cases. Surg Endosc (2012) 26(9):2687–94. doi: 10.1007/s00464-012-2227-2 22476831

[60] Qu R Tu D Hu S Wang Q Ping W Hao Z. Electromagnetic Navigation Bronchoscopy-Guided Microwave Ablation Combined With Uniportal Video-Assisted Thoracoscopic Surgery for Multiple Ground Glass Opacities. Ann Thorac Surg (2022) 113(4):1307–15. doi: 10.1016/j.athoracsur.2021.04.061 33964257

